# Reconsidering “Critical” Bone Loss in Shoulder Instability: 17-Year Follow-Up Study following Arthroscopic Bankart Repair

**DOI:** 10.1155/2024/5598107

**Published:** 2024-01-31

**Authors:** Lawrence Chun-Man Lau, Wai-Wang Chau, Randy Ng, Jonathan Patrick Ng, Elvis Chun-Sing Chui, Michael Tim-Yun Ong, James Francis Griffith, Patrick Shu-Hang Yung

**Affiliations:** ^1^Department of Orthopaedics and Traumatology, Faculty of Medicine, The Prince of Wales Hospital, Chinese University of Hong Kong, Shatin, Hong Kong SAR, China; ^2^Department of Imaging and Interventional Radiology, Faculty of Medicine, The Prince of Wales Hospital, Chinese University of Hong Kong, Shatin, Hong Kong SAR, China

## Abstract

**Background:**

Glenoid bone loss is a risk factor leading to the failure of arthroscopic Bankart repair. While 20–25% glenoid bone loss has long been considered the level to necessitate bony augmentation, recent studies indicate that 13.5% has a “subcritical” glenoid bone loss level, which is associated with decreased short- and medium-term functional scores. Few researchers worked on the long-term effect of “subcritical” or even less severe degrees of glenoid bone loss on redislocation rates and functional outcomes after arthroscopic Bankart repair. This study aimed to evaluate the effect of subcritical or less severe glenoid bone loss on redislocation rates and function after arthroscopic Bankart repair.

**Methods:**

A patient cohort who had undergone computed tomography (CT) of glenoid bone loss and arthroscopic Bankart repair over 15 years ago was reviewed. Western Ontario Shoulder Instability (WOSI) score, Single Assessment Numeric Evaluation (SANE) score, redislocation after operation, mechanism of recurrence, and revision details were reviewed.

**Results:**

Seventy-five patients were reassessed 17.6 ± 1.9 years following initial surgery. The age at enrolment was 26.8 ± 8.3 years. Twenty-two (29%) patients of the 75 patients had a redislocation on long-term follow-up, though this was not related to glenoid bone loss severity. The impaired functional score was found in patients with initial glenoid bone loss of 7% or more on long-term follow-up: WOSI (physical symptoms): 0.98 ± 2.00 vs 2.25 ± 4.01, *p*=0.04 and WOSI (total): 0.79 ± 1.43 vs 1.88 ± 3.56, *p*=0.04.

**Conclusions:**

At a mean of 17.5 years following arthroscopic Bankart repair, redislocation occurs in over a quarter of 75 patients, and they are not related to initial glenoid bone loss severity. Impaired functional outcome is apparent in patients with initial glenoid bone loss of >7%, though this impairment does not seem sufficiently severe to warrant an alternative treatment approach.

## 1. Introduction

Anterior glenoid bone loss is commonly associated with shoulder dislocation [1, 2]. Glenoid bone loss is a significant risk factor in the failure of arthroscopic Bankart repair [1, 3]. The degree of “critical” bone loss that warrants conversion to a bone augmentation procedure to address the bone defect has long been considered as greater than 20% to 25%, as more severe degree of glenoid bone loss increases the likelihood of redislocation following arthroscopic Bankart repair [2, 4, 5]. More recently, it was shown that “subcritical” (13.5% to 20%) glenoid bone loss was associated with a deterioration in the quality of life consistent with an unacceptable outcome [6]. Applying mathematical modeling, it has also been recently shown that anterior glenoid rim bone loss contributes most to the loss of shoulder stability [7]. It is, therefore, logical to think that, following arthroscopic Bankart repair in patients with subcritical, or ever lesser degree of bone loss, the repaired capsular tissue may have to withstand a higher pressure than the original capsulolabral tissue as some of the support provided by the bony glenoid rim has been lost [7]. Potentially, this repaired capsular tissue may attenuate over time leading to lower functional outcomes due to microinstability.

The concept of subcritical bone loss poses a treatment dilemma while arthroscopic Bankart repair is a more straightforward and safer operation, and it does carry a potentially higher risk of redislocation while bone augmentation procedures are more complex procedures although they may have a lower likelihood of recurrent dislocation [8]. In 2008, our group also highlighted the significance of subcritical bone loss in 218 patients with shoulder dislocation showing that “beyond a critical level of 13.4% glenoid bone loss, the number of dislocations experienced rose steeply from six to 10 dislocations” [9]. This study cohort also helped to formulate the Griffith Index, which was the first imaging-based study to explore the quantification of bone loss in anterior shoulder dislocation [9, 10]. Some of these study patients underwent arthroscopic Bankart repair subsequently. This allowed us to investigate the long-term redislocation risk and functional outcome following arthroscopic Bankart repair in patients with subcritical or lesser degrees of glenoid bone loss. We hypothesized that long-term functional outcomes would be negatively affected at glenoid bone loss levels below those previously reported as significant, irrespective of subsequent failure.

## 2. Materials and Methods

**Figure 1:**
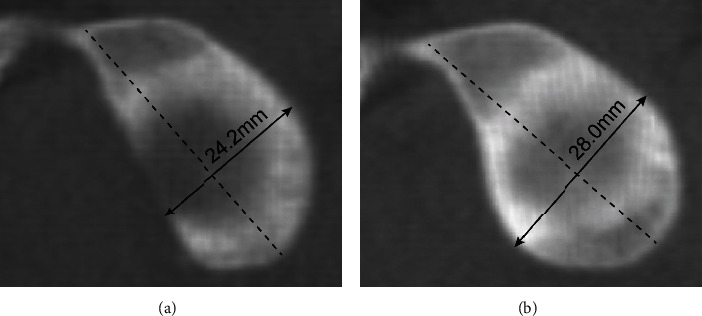
Measurement of glenoid bone loss in the dislocated shoulder in comparison to the contralateral unaffected side (Griffith Index). On the affected side (a), the maximum width at right angles to the long axis is 24.2 mm. On the unaffected side (b), the maximum width of the glenoid is 28.0 mm. The difference (28 mm − 24.2 mm) is 3.8 mm. As 3.8 mm/28.0 mm × 100 = 13.6%, this patient has 13.6% glenoid bone loss.

The study received ethics approval from our Institutional Ethics Review Committee (ethics approval number: 2022.019). The study design was referenced to the study conducted by Shaha and colleagues and was in accordance with the Declaration of Helsinki [6]. We followed up on the original cohort with anterior shoulder dislocation, and computed tomography (CT) scans performed between 2000 and 2006 [9, 10]. Among them, some have fulfilled the indication for arthroscopic Bankart repair with persistent symptoms of instability and/or apprehension sufficient to limit activity following initial conservative management and have undergone the surgery. All patients had preoperative magnetic resonance imaging (MRI) to confirm an anterior labral tear with or without bone loss (cohere to clinical examinations). Inclusion criteria included glenoid bone loss of less than 25% and nonengaging Hill–Sachs lesions. Exclusion criteria included (1) any patient with a concomitant procedure or diagnosis other than anterior shoulder instability, (2) extension of labral tear beyond the traditional Bankart lesion, (3) glenoid bone loss more than or equal to 25% bone loss, (4) engaging Hill-Sachs lesions, (5) multidirectional instability, (6) hyperlaxity with Beighton score ≥4/9, or (7) any patient who had undergone prior operative intervention to address glenohumeral instability of the affected shoulder. Preoperative CT examinations were performed to quantify the amount of glenoid bone loss as previously described [9, 10]. Multidetector CT examination of both shoulders was undertaken with the scan plane extending from the acromion to just below the glenoid, and the patient's arms were positioned by the chest wall. Double oblique reconstructions of each glenoid provided oblique sagittal images en-face to the glenoid articular surface (Advantage Windows, version 4.2, GE Healthcare) [9–11]. Glenoid bone loss was measured on CT using the Griffith Index (Figure 1) [9–11]. The maximum width at the mid-portion of the inferior glenoid was measured at right angles to the long axis of the glenoid. This was compared with the contralateral unaffected side to provide the degree of glenoid bone loss in mm (%). Investigator (J.G.) with 35 years of experience in analyzing CT examinations performed all CT reconstructions and measurements, with intraobserver agreement of 0.958 for glenoid width and 0.790 for glenoid length measurements reported in our series [9].

For patients who subsequently underwent isolated arthroscopic Bankart repair in our tertiary hospital between 2000 and 2006, case records were accessed through the hospital's electronic medical record system. Arthroscopic Bankart repair was operated by three sports surgeons using standard procedures. Surgeries were performed with the patients under general anesthesia in the lateral position with examination under anesthesia performed before starting the operation. In each case, a diagnostic shoulder arthroscopy from the posterior portal was performed first to confirm the diagnosis of isolated anterior instability leading to the Bankart lesion. Another two standard portals (anterior and anterosuperior) were then established. Labrum was released and mobilized with a glenoid rim prepared to obtain a bleeding surface. Arthroscopic Bankart repair was then performed with three or more suture anchors (2.9 mm; JuggerKnot®, Biomet Inc., Warsaw, IN, USA) inserting over 3–6 o'clock position fixing the labrum to the glenoid with emphasis on the capsular shift to retension the inferior and middle glenohumeral ligaments. All patients followed a standardized rehabilitation protocol comprising four weeks of immobilization with a shoulder immobilizer and abduction pillow together with supervised physical therapy. Therapy began with an active range of motion and activities of daily living on the operated arm. Strengthening was delayed until three months after surgery, and clearance to return to full activity was granted no sooner than six months after surgery.

In 2022, all eligible patients were contacted, and questionnaires were administered at interviews. Patients completed the Western Ontario Shoulder Instability Index (WOSI) questionnaire and Single Assessment Numeric Evaluation (SANE) with the aid of a trained assessor (R.N.) who was blinded to the study hypothesis. WOSI is a validated disease-specific assessment tool designed for shoulder instability, which is more responsive to change than other commonly used questionnaires [12]. SANE is a global rating scale of overall outcome from 0 to 100 points with a score of 100 representing the best possible outcome [13]. A number of redislocations or subluxations after the operation, mechanism of recurrence, and surgical revision details were reviewed through the electronic hospital record system with further validation from patients during an interview.

### 2.1. Statistical Analysis

Descriptive characteristics of baseline characteristics and glenoid bone loss were described using mean, standard deviation, and range (minimum and maximum). Sensitivity testings were carried out to compare different glenoid bone loss cut-off percentages with (i) redislocation after first operation, (ii) frequency of redislocation after first operation, and (iii) further operation after first operation. Initial glenoid bone loss cut-off values of >13.5%, ≤13.5%, 12%, 8%, 7%, and 6% were evaluated. Redislocation frequency, WOSI, and SANE scores were evaluated for these glenoid bone loss levels. Receiver operating curve (ROC) analyses on WOSI and SANE for glenoid bone loss cut-off values of 8%, 7%, and 6% were performed, and area under curve (AUC) values were summarized. Sensitivity and specificity on the optimum WOSI and SANE cut-off value were compared to decide the best possible prediction. All statistical analyses were carried out using IBM SPSS version 28 (IBM Corp. Released 2021. IBM SPSS Statistics for Windows, Version 28.0. Armonk, NY: IBM Corp).

## 3. Results

Among the 236 shoulders comprising the original cohort, 163 (69%) patients underwent arthroscopic Bankart repair, 17 (7.2%) underwent other types of operation, and 56 (24%) were treated conservatively (Table 1). Among the 163 patients who had arthroscopic Bankart repair, 75 patients were contactable, interviewed, and finished all clinical assessments. Age at enrolment (patients who underwent arthroscopic Bankart repair between 2000 and 2006) was 26.8 ± 8.3 years with current age (invited in 2022) 44.8 ± 8.5 years (Table 2). Among the 75 patients, 88% (*N* = 66) were male. Results from logistic regression modeling showed that neither younger age and sex nor combining age and sex was a risk factor for any incidence of redislocation after the first surgery (Table 3). The average follow-up was 17.6 ± 1.9 years.

### 3.1. Bone Loss Related to Redislocation

The average number of dislocations in these 75 patients before operation was 5.8 ± 6.5. Average glenoid bone loss was 9.0% ± 6.9%. Twenty-one (28%) patients of the 75 patients had glenoid bone loss >13.5%. No surgical complications occurred. Fifty-three (71%) patients of the 75 patients did not have further dislocation. In the remaining 22 (29%) patients with redislocation, this was due to trauma (fall from height, direct contusion, or fall on an outstretched hand) in all cases with the number of redislocations being 1.6 ± 0.7. Most redislocations required no further treatment (Table 4). Only 3 (13%) of the 22 patients with redislocation required a second operation (revision Bankart repair *N* = 2 and Latarjet procedure *N* = 1). No statistically significant glenoid bone loss threshold was identified to predict redislocation (Table 5).

### 3.2. Bone Loss Related to Functional Outcome

There was a trend of lower SANE with increasing severity of glenoid bone loss, but statistical significance had not been reached. No significant difference in WOSI at the subcritical bone loss of 13.5% (*p*=0.68) was found. However, a statistically significant difference did exist at glenoid bone loss cut-off of 7% for WOSI (physical symptoms): (0.98 ± 2.00 vs 2.25 ± 4.01, *p*=0.04) and WOSI (total): (0.79 ± 1.43 vs 1.88 ± 3.56, *p*=0.040) (Table 6).

Based on impaired WOSI (physical symptoms) and WOSI (total) at 7% glenoid bone loss, further ROC analyses using cut-off values at 8%, 7%, and 6% (i.e., plus or minus 1% at 7%) were tested. The best AUC was still found to be at a cut-off value of 7% for WOSI (physical symptoms) (AUC = 0.568) and WOSI (total) (AUC = 0.565). Further regression analysis was performed with adjustment for age and gender yielded similar findings.

### 3.3. Intraobserver Reliability

Intraobserver agreement was performed in our previous study, which was 0.958 for glenoid width and 0.790 for glenoid length measurements [9].

## 4. Discussion

This study demonstrated that arthroscopic Bankart repair, with or without subcritical bone loss, produces satisfactory long-term clinical outcomes. Over two-thirds of patients did not have further dislocation in an average of 17.5 years after surgery. In the operated cohort of which the patients are now 40 to 50 years old, their functional outcomes in terms of SANE and WOSI scores are satisfactory. Glenoid bone loss of 7% or more, here we described as “minimal critical” bone loss, relates to a lower functional outcome referenced by lower WOSI (physical symptoms) and WOSI (total) scores with statistical significance. Although we notice the relationship between lower SANE scores and increasing severity of glenoid bone loss, statistical significance has not been reached. The statistical insignificance shows that the functional outcome in patients with “minimal critical” glenoid bone loss is generally promising.

The good long-term clinical outcomes after arthroscopic Bankart repair in our cohort are comparable to other studies. In a recent systematic review, 69% of patients did not suffer further dislocation after arthroscopic Bankart repair on a mean follow-up period of 12.5 years [14]. Our satisfactory long-term outcome following arthroscopic Bankart repair can be explained by two reasons. First, our patient cohort may be relatively sedentary (low physical activity level) in their teenage and adulthood [15, 16]. Second, the composition of our cohort includes patients with different physical activity levels, namely, nonathletes (civilians), recreational athletes, and elite athletes. Despite their different physical activity levels at younger ages, their intensity and duration inevitably reduce with advancing age. The chance of joint dislocation is, therefore, reduced as a result of reduced sports participation in this long period of 17 years. The underlying reason for the reduced chance of dislocation with advancing age is reported as a result of the reduction in elasticity of the shoulder capsule and surrounding tissues [17, 18].

**Figure 2:**
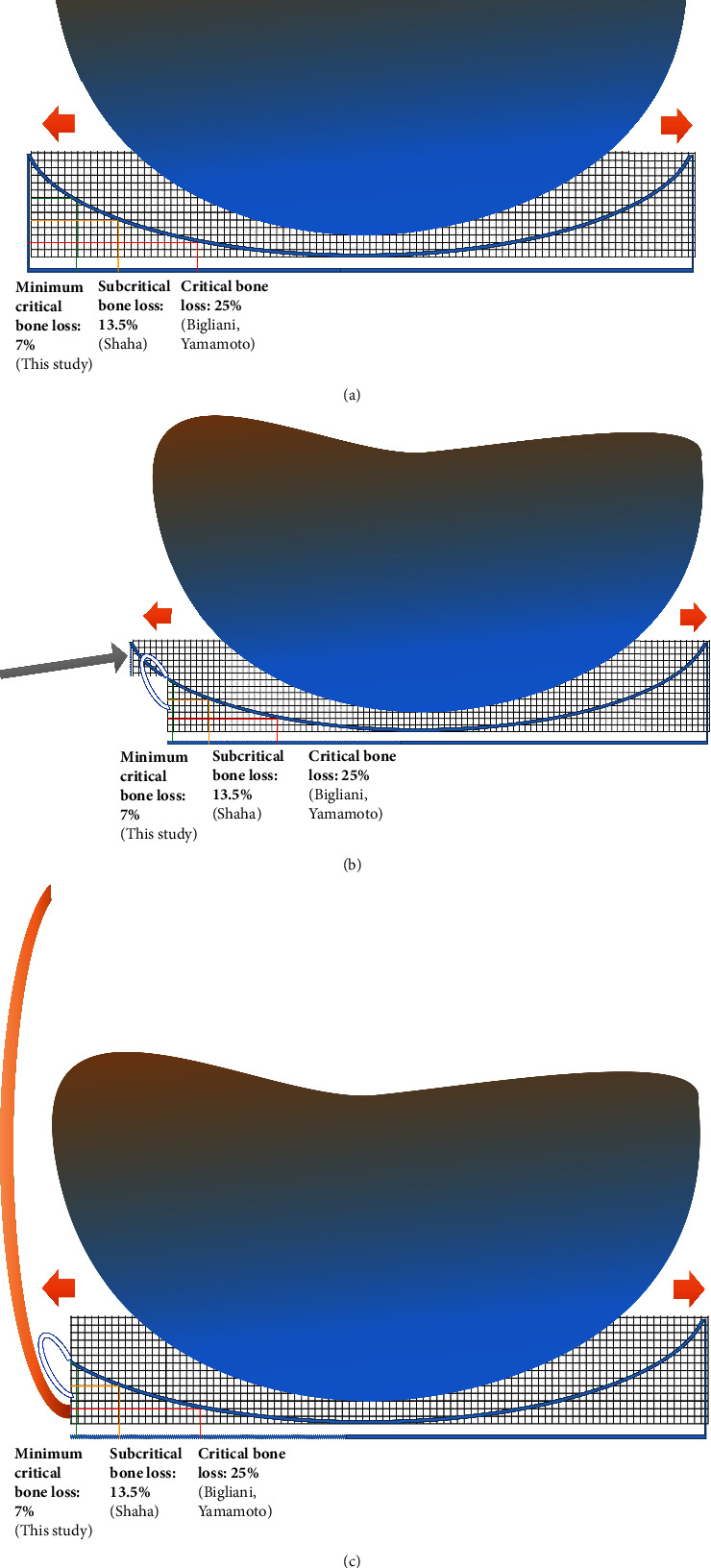
Graphical representation of minimal critical, subcritical, and critical bone loss concepts. (a) Hemisphere represents humeral head and concavity represents glenoid cavity which is drawn with gridlines of one hundred squares (100%). Minimal critical bone loss, subcritical bone loss, and critical bone loss are represented by green, yellow, and red lines, respectively. A nonlinear relationship between glenoid cavity depth and glenoid width loss is observed. (b) In arthroscopic Bankart repair in patients with minimal critical bone loss, the repaired capsulolabral tissue (represented by a blue-white-blue oval curve) replaces the glenoid bony rim (as shown by the grey arrow). This reparative tissue is likely to be under higher pressure than capsulolabral tissue in a normal position which has the additional support of a normal glenoid bony rim. (c) With aging, the likelihood of recurrent shoulder dislocations reduces due, in part, to a decrease in elasticity of the capsular and extracapsular soft tissues (orange curve) [17, 18].

“Critical” glenoid bone loss of 25% or more was reported to be associated with a high rate of arthroscopic Bankart repair failure. “Subcritical” glenoid bone loss of 13.5% or greater was also reported to be associated with a lower functional short-term outcome in high-demand individuals [4–6]. This study shows that glenoid bone loss of 7% or more (“minimal critical”) represents another level that is associated with mild appreciable long-term symptoms. This can be explained by a recently published finding by Moroder et al. in which nonlinear loss in stability with progressive glenoid bone loss was observed [7]. Due to the concave shape of the glenoid, the decrease in height of glenoid rim and the reduction in glenoid width exhibit an inverse exponential relationship as shown in Figure 2 [7, 19]. These observations try to explain that the most outer located glenoid rim contributes more to stability than the relatively more centrally located part [7]. Consequently, in arthroscopic Bankart repair, the repaired capsulolabral tissue in conjunction with glenoid bone loss is likely to be under higher pressure than the capsulolabral tissue in its original position without any bone loss, as now it is replacing the most outer located glenoid rim (Figure 2(a)). This increased pressure may lead to greater capsulolabral attenuation over time giving rise to microinstability symptoms without frank dislocation (Figure 2(b)). Importantly, this concept is in line with the recent publications suggesting that the increased concavity provided by thicker cartilage at the glenoid rim contributes decisively and significantly to the stability of the shoulder [19, 20]. In other words, the repaired capsulolabral tissue is not only replacing the glenoid bony rim that is shown on CT but also replacing the thicker cartilage at the glenoid rim. Therefore, the repaired capsulolabral tissue is under higher pressure than in its native position with the presence of a glenoid osteochondral rim.

This study also shows that in patients with glenoid bone loss of less than 7%, surgical strategies aiming to restore glenoid bone loss are not likely to provide added benefit over arthroscopic Bankart repair as there is no demonstrable difference in long-term functional outcome in patients who have <7% glenoid bone loss at the time of initial surgery. This is relevant as more, new, minimally invasive surgical procedures such as an arthroscopic iliac crest or scapular spine bone graft procedure are proposed to treat a lesser degree of glenoid bone loss [21–25]. This study shows that a simple arthroscopic Bankart repair alone suffices for this group of patients. In other words, while “critical” and “subcritical” bone loss are thresholds to perform bone augmentation procedures, “minimal critical” bone loss signifies a level that bone augmentation procedure is not necessary based on long-term clinical evidence.

The impairment in WOSI (physical symptoms and total) scores at a minimal critical bone loss level of 7% or more as shown in this study most likely reflects microinstability symptoms. This microinstability symptom has also been described in shoulders with Bankart lesions having clinically recurrent and painful micromotion without a history of dislocation [26]. This group of patients is similar to our cohort with minimal critical bone loss in presentation as both of them have mild symptoms without frank dislocation [26]. The differences in WOSI scores (physical symptoms and total domains) reported in our study are approximately close to the reported minimal clinically important difference (MCID). As a result, we term the glenoid bone loss of 7% or more as “minimal critical” based on our results [27]. Although being mentioned above that the repaired capsulolabral tissue has likely been attenuated over time due to higher pressure, additional age-related factors, such as loss of elastic fibers, would also have taken part due to aging and mitigate the microinstability effect (Figure 2(c)) [17]. Further advanced imaging analysis study on exploring elastic fiber content and structure integrity of Bankart repair at the same time is needed to evaluate this observation.

There are some limitations in this study. First, loss to follow-up is inevitable due to the study nature and long follow-up period. Our patients are keen on continuing their follow-up at our tertiary hospital because of our semifree healthcare policy which favors patients to do all clinical checkups, surgery, and postsurgery follow-up in public (government-funded) hospitals (as of our tertiary hospital). Therefore, we can notice the changes in their conditions, e.g., redislocation and pain, quickly and take prompt necessary actions [28]. Therefore, reasons for loss to follow-up are commonly due to (1) relocation or emigration and (2) symptom-free leading to nonresponsiveness to invitation. Checking the baseline characteristics and percentage of glenoid bone loss between patients who completed the study and patients who lost to follow-up, both showed similar results (Table 1). That implies no effect on data skewness after excluding those lost to follow-up. Second, the study cohort had the assessments and surgeries performed between 2000 and 2006, and the data helped to formulate the Griffith Index, which was the earliest imaging-based glenoid bone loss measurement method [9–11]. The formulation of the Griffith Index was earlier than the development of a well-validated imaging-based Hill–Sachs lesion size measurement method [29], before the glenoid track concept proposed in 2007 [30], and before the arthroscopic Bankart repair with remplissage described in 2008 [31]. We, however, excluded cases with engaging Hill–Sachs lesions because we did recognize the risk of engaging Hill–Sachs lesions described by Burkhart and De Beer in 2000 [4]. Although we did not perform the remplissage technique in this cohort, the results were satisfactory and comparable to other cohorts [14]. It is possible that avoiding engaging Hill-Sachs lesion has contributed significantly to the long-term successful rate [4]. Our study design is the same as the landmark “subcritical” bone loss study by Shaha et al. in 2018, in which this study also did not report on Hill–Sachs lesion in their cohort [6]. The relationship between “subcritical” bone losses with Hill–Sachs lesions was recently investigated by Yamamoto et al. who further developed the “glenoid track” concept into “central-track” and “peripheral-track” [32]. Due to the recency of this concept, they could not be investigated in our historical cohort but the interaction between “minimal critical” bone loss and “peripheral-track” may represent a future research direction.

In conclusion, arthroscopic Bankart repair is a safe surgical procedure with a satisfactory long-term outcome in shoulder dislocation patients with or without “subcritical” glenoid bone loss. A “minimal critical” glenoid bone loss of 7% or greater at the time of surgery is defined and that “minimal critical” glenoid bone loss is associated with mild long-term symptoms, reflected by lower WOSI (physical symptoms) and WOSI (total) scores. The degree of functional impairment does seem to be clinically acceptable as reflected by satisfactory long-term SANE scores.

## Figures and Tables

**Table 1:** Study flowchart.

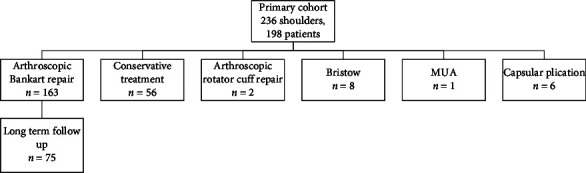

MUA: manipulation under anesthesia.

**Table 2:** Baseline demographics and glenoid bone loss (%) for 75 patients in this study and patients lost to follow-up.

	Included patients (*n* = 75)	Not included (*n* = 161)	*p* value
Age (current)	44.8 ± 8.5	45.3 ± 8.4	0.65
Sex			
Male	66 (88%)	137 (85%)	0.69
Female	9 (12%)	24 (15%)	
Glenoid bone loss (%)	7.7 ± 8.3	7.8 ± 6.9	0.95

**Table 3:** Logistic regression analysis of the effects of age and sex on any incidence of redislocation after the first surgery.

	Age (ref: >40)	Sex (ref: male)	*r * ^2^	Wald	OR (95% CI)	*p* value
Model 1	✓		0.02	0.77	1.85 (0.47–7.35)	0.38
Model 2		✓	0.12	0.00	0.00 (0.00–0.00)	1.00
Model 3			0.14			0.10
	✓			0.75	1.86 (0.46–7.61)	0.39
		✓		0.00	0.00 (0.00–0.00)	1.00

Dependent variable: any redislocation after the first surgery. OR: Odds ratio. CI: Confidence interval.

**Table 4:** Incidences of further dislocations after the first OT (*N* = 75).

Dislocation after first OT	22 (30.1)
Number of dislocations after first OT	
0	51 (69.9)
1	11 (15.1)
2	8 (11.0)
3	3 (4.1)
Unknown	2
Dislocation after the second OT	
No	75

**Table 5:** Redislocation related to glenoid bone loss of 13.5%, 12%, 8%, 7%, and 6% (*n* = 75).

Glenoid bone loss (%)	Redislocation after the 1st OT			Further dislocations after the 1st OT					Further operations after the 1st OT		
	Yes	No	*p* value	0	1	2	3	*p* value	Yes	No	*p* value
All	22 (29.3)	53 (70.7)	N/A	53 (70.7)	11 (14.7)	8 (10.0)	3 (4.0)	N/A	10 (13.3)	65 (86.7)	N/A

≤13.5	7 (33.3)	14 (66.7)	0.78	14 (66.7)	6 (28.6)	1 (4.8)	0	0.11	2 (9.5)	19 (90.5)	0.72
	(31.8)	(26.4)		(26.4)	(54.5)	(12.5)			(20.0)	(23.9)	

>13.5	15 (27.8)	39 (72.2)		39 (72.2)	5 (9.3)	7 (11.0)	3 (5.6)		8 (14.8)	46 (85.2)	
	(68.2)	(73.6)		(73.6)	(45.5)	(87.5)	(100.0)		(80.0)	(70.8)	

≤12	8 (32.0)	17 (68.0)	0.79	17 (68.0)	6 (24.0)	1 (4.0)	1 (4.0)	0.28	3 (12.0)	22 (88.0)	1.00
	(36.4)	(32.1)		(32.1)	(54.5)	(12.5)	(33.3)		(30.0)	(33.8)	

>12	14 (28.0)	36 (72.0)		36 (72.0)	5 (10.0)	7 (14.0)	2 (4.0)		7 (14.0)	43 (86.0)	
	(63.6)	(67.9)		(67.9)	(45.5)	(87.5)	(66.7)		(70.0)	(66.2)	

≤8	11 (28.9)	27 (71.1)	1.00	27 (71.1)	7 (18.4)	3 (7.9)	1 (2.6)	0.65	4 (10.5)	34 (89.5)	0.52
	(50.0)	(50.9)		(50.9)	(63.6)	(37.5)	(33.3)		(40.0)	(52.3)	

>8	11 (29.7)	26 (70.3)		26 (70.3)	4 (10.8)	5 (13.5)	2 (5.4)		6 (16.2)	31 (83.8)	
	(50.0)	(49.1)		(49.1)	(36.4)	(62.5)	(66.7)		(60.0)	(47.7)	

≤7	13 (30.2)	30 (69.8)	1.00	30 (69.8)	8 (18.6)	4 (9.3)	1 (2.3)	0.58	4 (9.3)	39 (90.7)	0.31
	(59.1)	(56.6)		(56.6)	(72.7)	(50.0)	(33.3)		(40.0)	(60.00)	

>7	9 (28.1)	23 (71.9)		23 (71.9)	3 (9.4)	4 (12.5)	2 (6.3)		6 (18.8)	26 (81.3)	
	(40.9)	(43.4)		(43.4)	(27.3)	(50.0)	(66.7)		(60.0)	(40.0)	

≤6	15 (33.3)	30 (66.7)	0.44	30 (66.7)	8 (17.8)	6 (13.3)	1 (2.2)	0.45	5 (11.1)	40 (88.9)	0.51
	(68.2)	(56.6)		(56.6)	(72.7)	(75.0)	(33.3)		(50.0)	(61.5)	

>6	7 (23.3)	23 (76.7)		23 (76.7)	3 (10.0)	2 (6.7)	2 (6.7)		5 (16.7)	25 (83.3)	
	(31.8)	(43.4)		(43.4)	(27.3)	(25.0)	(66.7)		(50.0)	(38.5)	

**Table 6:** WOSI scores and SANE scores related to glenoid bone loss of 13.5%, 12%, 8%, 7%, and 6% (*n* = 75).

Bone loss (*N*)	WOSI (%)										SANE (%)	
	Physical symptoms		Sports/Recreation/Work		Lifestyle		Emotion		Total			
	Mean	*p* value	Mean	*p* value	Mean	*p* value	Mean	*p* value	Mean	*p* value	Mean	*p* value
All (75)	1.52 ± 3.07	N/A	1.34 ± 3.22	N/A	1.07 ± 3.22	N/A	0.53 ± 2.63	N/A	1.25 ± 2.60	N/A	91.73 ± 9.78	N/A
≤13.5 (54)	1.10 ± 1.95	0.46	1.26 ± 3.32	0.73	0.95 ± 3.40	0.85	0.32 ± 1.46	0.66	1.05 ± 1.70	0.68	90.48 ± 10.24	0.49
>13.5 (21)	1.69 ± 3.41		1.55 ± 3.01		1.11 ± 3.25		0.62 ± 2.97		1.33 ± 2.88		92.22 ± 9.65	
≤12 (50)	1.08 ± 1.82	0.39	1.31 ± 2.80	0.96	0.80 ± 3.12	0.62	0.27 ± 1.33	0.54	1.00 ± 1.57	0.57	90.80 ± 9.54	0.56
>12 (25)	1.74 ± 3.53		1.36 ± 3.43		1.20 ± 3.36		0.67 ± 3.09		1.37 ± 2.99		92.20 ± 9.96	
≤8 (37)	1.11 ± 2.10	0.12	1.00 ± 2.43	0.18	0.72 ± 2.78	0.18	0.18 ± 1.08	0.12	0.86 ± 1.50	0.10	91.84 ± 9.26	0.46
>8 (38)	1.95 ± 3.80		1.69 ± 3.87		1.42 ± 3.71		0.90 ± 3.57		1.65 ± 3.35		91.62 ± 10.41	
≤7 (32)	0.98 ± 2.00	**0.04**	1.00 ± 2.38	0.16	0.64 ± 2.62	0.11	0.16 ± 1.02	0.11	0.79 ± 1.43	**0.04**	92.33 ± 8.95	0.27
>7 (43)	2.25 ± 4.01		1.80 ± 4.08		1.64 ± 3.95		1.04 ± 3.83		1.88 ± 3.56		90.94 ± 10.88	
≤6 (30)	1.16 ± 2.22	0.13	1.07 ± 2.41	0.21	0.83 ± 2.92	0.23	0.15 ± 0.99	0.10	0.92 ± 1.66	0.12	91.56 ± 9.52	0.43
>6 (45)	2.07 ± 4.00		1.75 ± 4.16		1.42 ± 3.75		1.11 ± 3.95		1.74 ± 3.56		92.00 ± 10.31	

Significant results are bolded.

## Data Availability

The dataset used to support the findings of this study is available from the corresponding author upon request.

## References

[1] Itoi E., Lee S. B., Berglund L. J., Berge L. L., An K. N. (2000). The effect of a glenoid defect on anteroinferior stability of the shoulder after bankart repair: a cadaveric study. *Journal of Bone and Joint Surgery American Volume*.

[2] Boileau P., Villalba M., Hery J. Y., Balg F., Ahrens P., Neyton L. (2006). Risk factors for recurrence of shoulder instability after arthroscopic Bankart repair. *The Journal of Bone and Joint Surgery*.

[3] Montgomery W. H., Wahl M., Hettrich C., Itoi E., Lippitt S. B., Matsen F. A. (2005). Anteroinferior bone-grafting can restore stability in osseous glenoid defects. *The Journal of Bone and Joint Surgery*.

[4] Burkhart S. S., De Beer J. F. (2000). Traumatic glenohumeral bone defects and their relationship to failure of arthroscopic Bankart repairs: significance of the inverted-pear glenoid and the humeral engaging hill-sachs lesion. *Arthroscopy: The Journal of Arthroscopic & Related Surgery*.

[5] Lo I. K., Parten P. M., Burkhart S. S. (2004). The inverted pear glenoid: an indicator of significant glenoid bone loss. *Arthroscopy: The Journal of Arthroscopic & Related Surgery*.

[6] Shaha J. S., Cook J. B., Song D. J. (2015). Redefining critical bone loss in shoulder instability: functional outcomes worsen with subcritical bone loss. *The American Journal of Sports Medicine*.

[7] Moroder P., Damm P., Wierer G. (2019). Challenging the current concept of critical glenoid bone loss in shoulder instability: does the size measurement really tell it all?. *The American Journal of Sports Medicine*.

[8] Shubert S. B. (2021). Editorial commentary: surgical treatment of shoulder instability with subcritical glenoid bone loss requires innovation: Bankart may risk significant recurrence and Latarjet may risk significant complications. *Arthroscopy: The Journal of Arthroscopic & Related Surgery*.

[9] Griffith J. F., Antonio G. E., Yung P. S. (2008). Prevalence, pattern, and spectrum of glenoid bone loss in anterior shoulder dislocation: CT analysis of 218 patients. *American Journal of Roentgenology*.

[10] Griffith J. F., Antonio G. E., Tong C. W., Ming C. K. (2003). Anterior shoulder dislocation: quantification of glenoid bone loss with CT. *American Journal of Roentgenology*.

[11] Griffith J. F., Yung P. S., Antonio G. E., Tsang P. H., Ahuja A. T., Chan K. M. (2007). CT compared with arthroscopy in quantifying glenoid bone loss. *American Journal of Roentgenology*.

[12] Kirkley A., Griffin S., McLintock H., Ng L. (1998). The development and evaluation of a disease-specific quality of life measurement tool for shoulder instability. *The American Journal of Sports Medicine*.

[13] Ladermann A., Denard P. J., Collin P., Ibrahim M., Bothorel H., Chih-Hao Chiu J. (2021). Single assessment numeric evaluation for instability as an alternative to the rowe score. *Journal of Shoulder and Elbow Surgery*.

[14] Murphy A. I., Hurley E. T., Hurley D. J., Pauzenberger L., Mullett H. (2019). Long-term outcomes of the arthroscopic bankart repair: a systematic review of studies at 10-year follow-up. *Journal of Shoulder and Elbow Surgery*.

[15] Huang W. Y., Wong S. H. S., Sit C. H. P. (2019). Results from the hong kong’s 2018 report card on physical activity for children and youth. *Journal of Exercise Science & Fitness*.

[16] Bauman A., Ainsworth B. E., Sallis J. F. (2011). The descriptive epidemiology of sitting. *American Journal of Preventive Medicine*.

[17] Castagna A., Cesari E., Gigante A., Di Matteo B., Garofalo R., Porcellini G. (2018). Age-related changes of elastic fibers in shoulder capsule of patients with glenohumeral instability: a pilot study. *BioMed Research International*.

[18] Szyluk K., Jasinski A., Niemiec P., Mielnik M., Widuchowski W., Koczy B. (2018). Male gender and age range 20-29 years are the most important non-modifiable risk factors for recurrence after primary post-traumatic shoulder dislocation. *Knee Surgery, Sports Traumatology, Arthroscopy*.

[19] Wermers J., Raschke M. J., Wilken M., Riegel A., Katthagen J. C. (2021). The anatomy of glenoid concavity-bony and osteochondral assessment of a stability-related parameter. *Journal of Clinical Medicine*.

[20] Souleiman F., Zderic I., Pastor T. (2022). Cartilage decisively shapes the glenoid concavity and contributes significantly to shoulder stability. *Knee Surgery, Sports Traumatology, Arthroscopy*.

[21] Boehm E., Minkus M., Moroder P., Scheibel M. (2021). Arthroscopic iliac crest bone grafting in recurrent anterior shoulder instability: minimum 5-year clinical and radiologic follow-up. *Knee Surgery, Sports Traumatology, Arthroscopy*.

[22] Xiang M., Yang J., Chen H. (2021). Arthroscopic autologous scapular spine bone graft combined with bankart repair for anterior shoulder instability with subcritical (10%-15%) glenoid bone loss. *Arthroscopy: The Journal of Arthroscopic & Related Surgery*.

[23] St Jeor J. D., Li X., Waterman B. R. (2023). Editorial commentary: glenoid reconstruction with autologous tricortical iliac crest represents an alternative to bankart repair and remplissage for anterior shoulder instability with subcritical bone loss. *Arthroscopy: The Journal of Arthroscopic & Related Surgery*.

[24] Wu D., Zhou Z., Song W. (2023). Arthroscopic autologous iliac crest grafting results in similar outcomes and low recurrence compared to remplissage plus bankart repair for anterior shoulder instability with bipolar bone defects. *Arthroscopy: The Journal of Arthroscopic & Related Surgery*.

[25] Griffith R., Tibone J. E., McGarry M. H., Adamson G. J., Lee T. Q. (2023). Biomechanical comparison of open bankart repair vs. Conjoint tendon transfer in a 10% anterior glenoid bone loss shoulder instability model. *Journal of Shoulder and Elbow Surgery*.

[26] Kim S. C., Kim K. H., Park J. H. (2022). Microinstability characterised by small and easily overlooked anterior labral or hill-sachs lesions can be managed with arthroscopic anterior labral repair. *Knee Surgery, Sports Traumatology, Arthroscopy*.

[27] Park I., Lee J. H., Hyun H. S., Lee T. K., Shin S. J. (2018). Minimal clinically important differences in rowe and western ontario shoulder instability index scores after arthroscopic repair of anterior shoulder instability. *Journal of Shoulder and Elbow Surgery*.

[28] Xiong X., Li V. J., Huang B., Huo Z. (2022). Equality and social determinants of spatial accessibility, availability, and affordability to primary health care in hong kong, a descriptive study from the perspective of spatial analysis. *BMC Health Services Research*.

[29] Maio M., Sarmento M., Moura N., Cartucho A. (2019). How to measure a hill-sachs lesion: a systematic review. *EFORT Open Reviews*.

[30] Yamamoto N., Itoi E., Abe H. (2007). Contact between the glenoid and the humeral head in abduction, external rotation, and horizontal extension: a new concept of glenoid track. *Journal of Shoulder and Elbow Surgery*.

[31] Purchase R. J., Wolf E. M., Hobgood E. R., Pollock M. E., Smalley C. C. (2008). Hill-sachs remplissage: an arthroscopic solution for the engaging hill-sachs lesion. *Arthroscopy: The Journal of Arthroscopic & Related Surgery*.

[32] Yamamoto N., Shinagawa K., Hatta T., Itoi E. (2020). Peripheral-track and central-track hill-sachs lesions: a new concept of assessing an on-track lesion. *The American Journal of Sports Medicine*.

